# Efficacy and safety of estradiol/dydrogesterone combined with escitalopram in the treatment of anxiety and depression in perimenopausal women: a randomized controlled trial

**DOI:** 10.3389/fphys.2026.1728736

**Published:** 2026-04-02

**Authors:** Shufang Wu, Guiying Zeng, Saisai Chen, Hongbin Chen, Senpei Xie, Jiabao Zhang, Yingchun Xiao, Nan Liu, Hui Chen, Ronghua Chen

**Affiliations:** 1 Department of Neurology, Fujian Medical University Union Hospital, Fuzhou, China; 2 Fujian Key Laboratory of Molecular Neurology, Fujian Medical University, Fuzhou, China; 3 Institute of Clinical Neurology, Fujian Medical University, Fuzhou, China; 4 Clinical Research Center for Precision Diagnosis and Treatment of Neurological Diseases of Fujian Province, Fuzhou, China; 5 Gynecology Department, Fujian Maternity and Child Health Hospital, Affiliated Hospital of Fujian Medical University, Fuzhou, China

**Keywords:** anxiety, depression, estradiol/dydrogesterone, escitalopram, perimenopausal women

## Abstract

**Background:**

To investigate the efficacy and underlying mechanisms of a combined therapy of estradiol/dydrogesterone and escitalopram in treating perimenopausal anxiety and depression.

**Methods:**

A total of 195 patients were randomized to receive escitalopram, estradiol/dydrogesterone, or combination therapy for 12 weeks. The primary efficacy endpoints were the changes in scores of the Hamilton Depression Rating Scale (HAMD) and Hamilton Anxiety Rating Scale (HAMA) at week 12 compared with the baseline.

**Results:**

Compared with the baseline, all groups reported a gradual decrease in Patient Health Questionnaire Somatic Symptom Scale (PHQ-15), the Patient Health Questionnaire Depression Scale (PHQ-9), Generalized Anxiety Disorder Scale (GAD-7), HAMD, and HAMA scores (P<0.001 for all), and a gradual increase in serum estradiol (E2) and 5-Hydroxytryptamine (5-HT) (P<0.001 for both), with the most pronounced changes in the Combined group (Comb group). In intergroup comparison, at weeks 4, 8, 12, the scores of PHQ-15, GAD-7, PHQ-9, HAMA, and HAMD were most reduced in the Comb group; At weeks 2, 4, 8, 12, E2 level was significantly higher in the Comb group and estradiol/dydrogesterone group (E/D group) than in the escitalopram group (ESC group); at weeks 4, 8, and 12, 5-HT level was significantly higher in the Comb group than in the E/D group; and at week 4 and 12, 5-HT level was significantly higher in the Comb group than in the ESC group.

**Conclusions:**

Estradiol/dydrogesterone plus escitalopram significantly improves anxiety, depression, and somatic symptoms in perimenopausal women. These findings provide clinical evidence of estradiol/dydrogesterone plus escitalopram regimen for the treatment of patients with perimenopausal anxiety and depression.

## 1 Introduction

Perimenopause is a period of significant physiological and psychological changes in women. During this period, in addition to symptoms such as menstrual disorders, hot flashes, and insomnia, perimenopausal women often experience significant emotional distress, particularly anxiety and depression (Liu et al., 2025). As compared with the perimenopausal period, these women are at an exponentially-increased risk for depression or recurrence of depression in the perimenopausal period (Bromberger and Kravitz, 2011; Badawy et al., 2024; Freeman et al., 2014; Bromberger et al., 2013),with a prevalence ranging from 45 to 68% (Horst et al., 2025). Perimenopausal depression is also associated with an increased risk of suicide, severely affecting quality of life and family harmony in perimenopausal women (Pérez-López et al., 2009). Therefore, it is crucial to initiate an early diagnosis and treatment of anxiety and depression in perimenopausal women.

The pathogenesis of perimenopausal anxiety and depression remains unknown. The current belief holds that the decrease of estrogen in this particular population plays a crucial role, as estrogen fluctuations are closely associated with perimenopausal anxiety and depression (Gordon et al., 2019; Joffe et al., 2020; Schmidt et al., 2015; Lozza-Fiacco et al., 2022). Available evidence suggests that estrogen fluctuations can induce the dysregulation of associated neurotransmitters, such as 5-Hydroxytryptamine (5-HT) and dopamine, and impact the hypothalamic-pituitary-adrenal axis, leading to perimenopausal anxiety and depression (Soares, 2014; Gordon et al., 2021). Other studies have documented that the variability of estradiol (E2) is associated with the severity of perimenopausal depression (Freeman et al., 2004). Meanwhile, estrogen replacement therapy has been demonstrated to improve anxiety and depression symptoms in perimenopausal women (Kulkarni et al., 2018; Lozza-Fiacco et al., 2022). In animal studies, estrogen can modulate the 5-HT pathway by regulating the synthesis and re-uptake of 5-HT and the number of its receptors (Sánchez et al., 2013; Bethea et al., 2000; Gundlah et al., 2002), which are closely associated with the development of anxiety and depression. However, it remains obscure whether the improvement in perimenopausal anxiety and depression is mediated by the change in serum E2 level or by that of serum 5-HT level.

Currently, the main pharmacological treatments for perimenopausal anxiety and depression are selective serotonin reuptake inhibitors (SSRIs) and serotonin-norepinephrine reuptake inhibitors (Maki et al., 2018). The SSRI treatment has been found to alleviate anxiety and depression in perimenopausal women, despite a slow onset of action and a low rate of remission (Maki et al., 2018). Some studies have confirmed the effectiveness of estrogen replacement therapy for perimenopausal anxiety and depression (Kulkarni et al., 2018; Lozza-Fiacco et al., 2022). Still some suggest that the use of estrogen as an adjunct to antidepressants may demonstrate a greater efficacy in alleviating perimenopausal depressive symptoms when compared with a monotherapy with either (Maki et al., 2018). However, no randomized controlled trials have been conducted to assess the efficacy of combining hormones and SSRIs in the treatment of perimenopausal depression.

Therefore, in the current study, a double-blind randomized controlled trial (RCT) was conceived to investigate whether estradiol/dydrogesterone plus escitalopram might produce a favorable efficacy in treating perimenopausal anxiety and depression; whether estradiol/dydrogesterone may enhance the therapeutic efficacy of escitalopram in the treatment of anxiety and depression in perimenopausal women; and whether the fluctuation in serum E2 and 5-HT levels plays a mediating role in the treatment of perimenopausal anxiety and depression. The findings may provide a clinical basis for optimizing the treatment of perimenopausal anxiety and perimenopausal depression.

## 2 Methods

### 2.1 Patients

From October 2021 to March 2023, this study recruited subjects with perimenopausal anxiety or perimenopausal depression who had received the inpatient and outpatient services of two institutions: Fujian Maternity and Child Health Hospital and Fujian Medical University Union Hospital.

Inclusion criteria were: (1) perimenopause as defined by the Stages of Reproductive Aging Workshop (Harlow et al., 2012); (2) intact uterus and ovaries; (3) no history of radiotherapy and/or chemotherapy; (4) no use of any drugs for the treatment of adverse perimenopausal symptoms in the past three months; (5) meeting the diagnostic criteria for depressive or anxiety disorders in the Diagnostic and Statistical Manual of Mental Disorders (DSM-5) of the American Psychiatric Association (American Psychiatric Association, 2013; 5th edition); with a score of ≥ 8 on the 17-item Hamilton Depression Rating Scale (HAMD-17) (Zimmerman et al., 2013),or a score of ≥ 14 on the 14-item Hamilton Anxiety Rating Scale (HAMA-14) (Hamilton, 1959), and (6) ability to complete questionnaires independently and sign the informed consent form, and willingness to participate in the study.

Exclusion criteria included: (1) the presence of other mental illnesses other than depressive and anxiety disorders; (2) severe physical diseases involving the heart, liver, kidneys, and respiratory system; (3) poor medication adherence [score < 6 on the 8-item Morisky Medication Adherence Scale (MMAS-8)] (Furue et al., 2015); (4) high suicide risk [score > 30 on the Suicide Assessment Scale (SUAS)] (Waern et al., 2010); and (5) being allergic to estradiol/dydrogesterone or escitalopram.

### 2.2 Study design

The study protocol was approved by the Ethics Committee of Fujian Maternity and Child Health Hospital (Ethics Batch No. 2021KLRD09018), which also applies to Fujian Medical University Union Hospital. Among the 221 patients with perimenopausal anxiety and perimenopausal depression initially recruited, 195 were finally enrolled for the study.

Patients were numbered according to the enrollment sequence and randomly grouped at a 1:1:1 allocation ratio according to the order of random numbers generated with the Statistical Products and Services Solutions 17.0 software (SPSS Inc., Chicago, IL, USA): escitalopram + placebo (ESC group), estradiol/dydrogesterone + placebo (E/D group), and estradiol/dydrogesterone plus escitalopram (Comb group). The ESC group received escitalopram (Bailot, Sichuan Kelun Pharmaceutical Co., Ltd.) at a dosage of 10 mg once daily. In case of adverse events (AEs), the dose was reduced to 5 mg once daily for 3 days and increased to 10 mg for maintenance therapy if the AEs were relieved. The placebo was an analogue to estradiol/dydrogesterone and administered in the same dosage regimen of estradiol/dydrogesterone. The E/D group received estradiol/dydrogesterone 1/10 (Solvay Pharmaceutical Co., Ltd.), which consists of a white tablet (estradiol 1mg) and a gray tablet (estradiol 1mg + dydrogesterone 10mg). Specifically, an initial 28-day course of one tablet daily involved the white tablet taken for the first 14 days and the gray tablet for the next 14 days; and a second course started from day 29. If the discomfort associated with estrogen insufficiency did not improve, the patients were given estradiol/dydrogesterone 2/10 (Solvay Pharmaceuticals Ltd.), composed of a brick red tablet (estradiol 2 mg) and a yellow tablet (estradiol 2 mg + dydrogesterone 10 mg), following the same dosage scheme. The placebo was an escitalopram analogue, given at a dosage of 10 mg once daily. The Comb group received estradiol/dydrogesterone and escitalopram simultaneously according to the above schema. The dosage form, color and taste of the placebo were consistent with the actual use of escitalopram or estradiol/dydrogesterone tablets. The trial comprised a 12-week active treatment phase. All participants first received a medication washout: 1 week for most agents and 2 weeks for previously-prescribed monoamine oxidase inhibitors (Andrade, 2022).

### 2.3 Baseline characteristics

At baseline, the collected data included education, age, body mass index (BMI), HAMA score, HAMD score, the Patient Health Questionnaire-15 (PHQ-15) score (Liao et al., 2016), PHQ-9 score (Spitzer et al., 1999), GAD-7 score (Spitzer et al., 2006), and levels of serum E2 and 5-HT. Post-treatment assessments (including the aforementioned scale and blood tests) were performed at weeks 2, 4, 8, and 12.

### 2.4 Outcomes

The primary efficacy outcomes were changes in HAMD and HAMA scores from baseline at week 12. The secondary outcomes included changes in PHQ-15 score, PHQ-9 score, GAD-7 score, and serum E2 and 5-HT levels during the 12-week treatment period.

### 2.5 Blood sample collection and testing

Fasting blood samples were collected prior to the treatment and at weeks 2, 4, 8, and 12 after the start of treatment. Serum concentrations of 5-HT and E2 were measured by enzyme-linked immunosorbent assay. The analytical procedures are detailed in the **Supplementary Material**.

### 2.6 Follow-up and AEs assessment

The follow-up period of this study extended for 12 weeks. HAMD, HAMA, PHQ-15, PHQ-9 and GAD-7 scales were used to assess the efficacy at weeks 2, 4, 8 and 12 after the treatment. Blood indices, renal function, liver function, blood pressure and electrolytes, and electrocardiogram were monitored during treatment. The Treatment Emergency Symptom Scale (TESS) was employed to gauge the severity of AEs (National Institute of Mental Health, 1985).

### 2.7 Statistical analysis

Statistical analyses were processed with the SPSS 27.0 software. The main efficacy results were analyzed by the intention-to-treat (ITT) method. The normality of data was checked by frequency histograms, Q-Q plots, and Kolmogorov-Smirnov test. Measurements were expressed as mean ± standard deviation (x ± s). The intergroup differences in baseline data were compared by one-way ANOVA. The inter- and intra-group comparisons of variables during follow-up were assessed by the generalized estimating equations (GEE). The impact of the fluctuation of the serum E2 and 5-HT levels on the mood improvement was evaluated by PROCESS macros. The association of serum E2 and 5-HT with HAMA and HAMD scores was respectively analyzed by Pearson’s partial correlation. A two-sided P value of less than 0.05 was considered statistically significant.

## 3 Results

### 3.1 Baseline information

**Figure 1:**
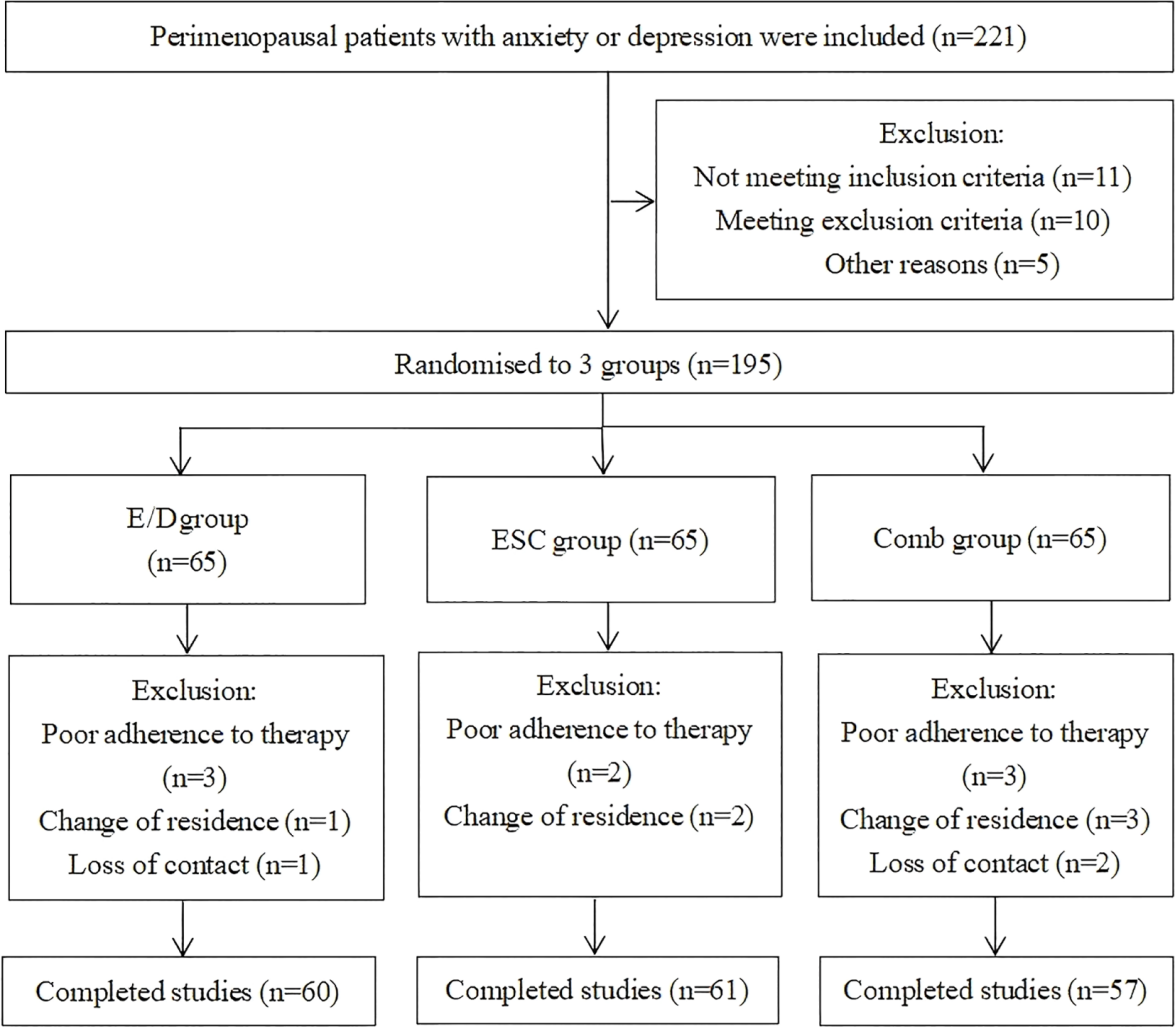
Flowchart of patient enrollment.

The 195 enrolled participants were randomly assigned to three groups, with 65 patients in each group (**Figure 1**). The three groups showed no significant differences in baseline characteristics (**Table 1**).

**Table 1:** Comparison of baseline data for the three groups.

Item	E/D group (n=65)	ESC group (n=65)	Comb group (n=65)	*P*
Age, years	49.58 ± 2.10	49.62 ± 2.23	49.78 ± 2.14	0.850
Education years, year	6.88 ± 3.76	6.94 ± 3.57	6.98 ± 3.39	0.985
BMI, kg/m^2^	23.56 ± 1.23	23.36 ± 1.67	23.33 ± 1.46	0.627
PHQ-15 Score	11.23 ± 1.75	11.52 ± 1.72	11.22 ± 1.81	0.533
GAD-7 Score	11.69 ± 1.81	12.02 ± 1.79	11.94 ± 2.05	0.595
PHQ-9 Score	11.45 ± 2.77	11.71 ± 2.69	11.11 ± 3.02	0.481
HAMD Score	12.66 ± 2.05	12.86 ± 2.34	12.54 ± 2.42	0.716
HAMA Score	20.06 ± 2.78	20.31 ± 2.92	20.08 ± 2.78	0.857
Serum E2, pg/ml	33.30 ± 3.15	34.22 ± 3.32	33.48 ± 3.68	0.262
Serum 5-HT, ng/ml	360.69 ± 25.15	356.96 ± 24.55	355.99 ± 21.27	0.492

E/D group as control group; ESC group:escitalopram group; Comb group: Combined group; Data are shown as mean ± standard deviation. BMI, Body Mass Index; PHQ-15, Patient Healthy Questionnaire-15; GAD-7, Generalized Anxiety Disorder-7; PHQ-9, Patient Health Questionnaire-9; HAMD, Hamilton Depression Rating Scale; HAMA, Hamilton Anxiety Scale; 5-HT, 5-hydroxytryptamine; E2, Estradiol;.

### 3.2 Primary endpoints

**Figure 2:**
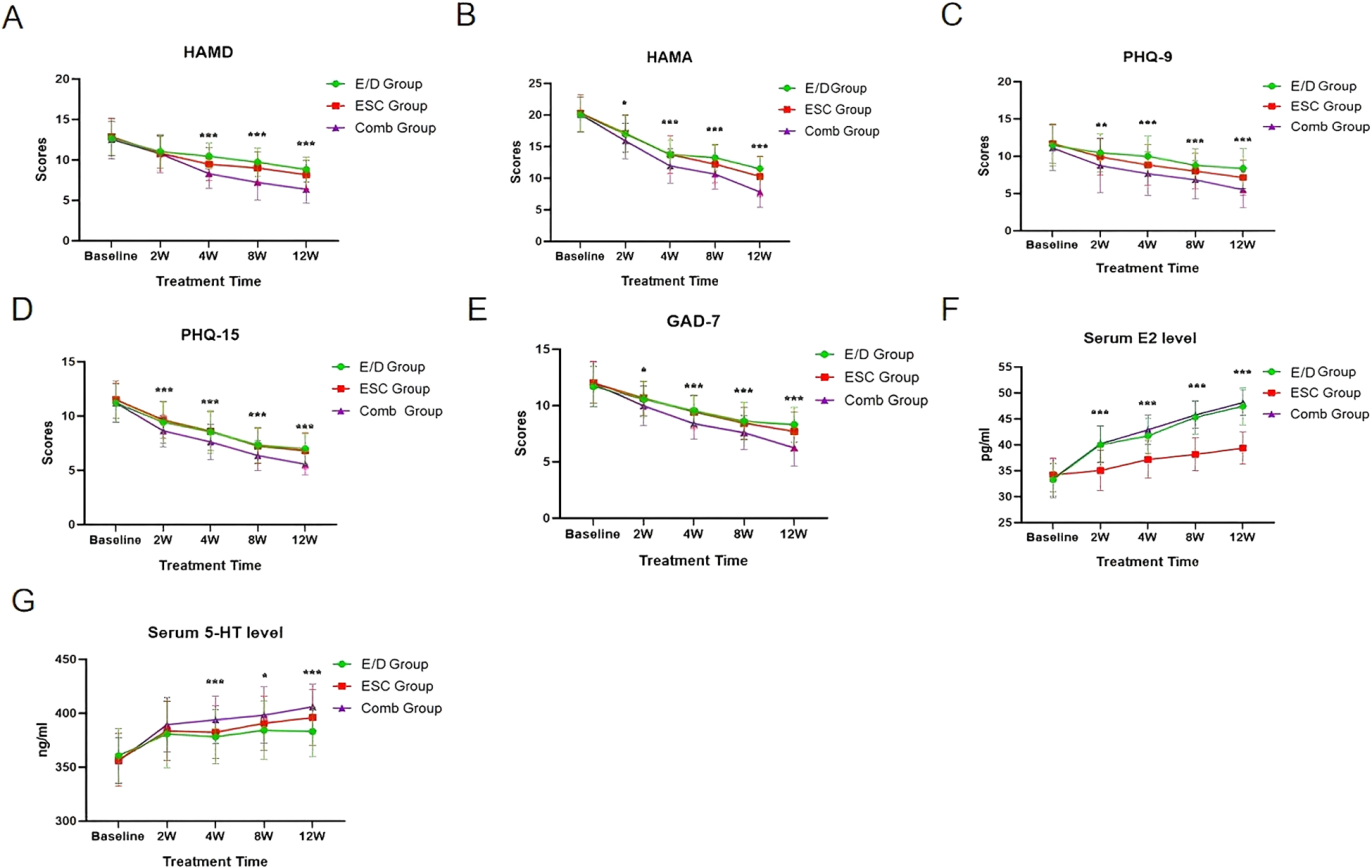
Changes in primary outcome indicators and secondary outcome indicators. **(A)** Comparison of HAMD scores among the three groups. **(B)** Comparison of HAMA scores among the three groups. **(C)** Comparison of PHQ- 9 scores among the three groups. **(D)** Comparison of PHQ- 15 scores among the three groups. **(E)** Comparison of GAD-7 score in the three groups. **(F)** Comparison of serum E2 levels among the three groups. **(G)** Comparison of serum 5-HT levels among the three groups. PHQ-15, Patient Healthy Questionnaire-15; GAD-7, Generalized Anxiety Disorder-7; PHQ-9, Patient Health Questionnaire-9; HAMD, Hamilton Depression Rating Scale; HAMA, Hamilton Anxiety Scale; 5-HT, 5-hydroxytryptamine; E2, Estradiol.

Changes in the primary endpoint indicators are shown in **Table 2** and **Figure 2**. As shown in **Table 2**, a significant interaction effect was evident between grouping and treatment duration for all outcome indicators (P<0.001). Compared with the baseline data, all three groups reported a gradual decrease in HAMD, and HAMA scores (P<0.001 for both), with the most significant change found in the Comb group.

**Table 2:** Changes in primary endpoints (HAMA and HAMD scores) from baseline.

Item	Time	Changes from Baseline (95%CI; P)			Inter-group difference (95%CI; P)			Group-by-timeInter-actionEffect
		E/D group	ESC group	Comb group	E/D groupvs ESC group	E/D groupvs*Comb group*	ESC groupvs Comb group	
HAMD Score	2W	-1.63 (-1.93, -1.33; **< 0.001**)	-2.03 (-2.52, -1.54; **< 0.001**)	-1.77 (-2.16, -1.38; **< 0.001**)	0.2 (-0.49, 0.89;0.568)	0.26 (-0.48, 1.00;0.487)	0.06 (-0.69, 0.81;0.873)	**< 0.001**
	4W	-2.2 (-2.48, -1.92; **< 0.001**)	-3.35 (-3.86, -2.85; **< 0.001**)	-4.23 (-4.68, -3.78; **< 0.001**)	0.95 (0.33, 1.58; **0.003**)	2.15 (1.57, 2.74; **< 0.001**)	1.2 (0.54, 1.86; **< 0.001**)	
	8W	-2.94 (-3.31, -2.57; **< 0.001**)	-3.86 (-4.34, -3.38; **< 0.001**)	-5.32 (-5.79, -4.85; **< 0.001**)	0.72 (0.09, 1.36; **0.026**)	2.51 (1.83, 3.18; **< 0.001**)	1.78 (1.08, 2.49; **< 0.001**)	
	12W	-3.85 (-4.33, -3.37; **< 0.001**)	-4.68 (-5.15, -4.20; **< 0.001**)	-6.18 (-6.68, -5.69; **< 0.001**)	0.63 (0.07, 1.19; **0.028**)	2.46 (1.91,3.01; **< 0.001**)	1.83 (1.25, 2.42; **< 0.001**)	
HAMA Score	2W	-3.03 (-3.45, -2.61; **< 0.001**)	-3.2 (-3.68, -2.72; **< 0.001**)	-4.17 (-5.00, -3.33; **< 0.001**)	-0.08 (-1.07, 0.92;0.880)	1.12 (0.15, 2.10; **0.024**)	1.2 (0.21, 2.19; **0.017**)	**< 0.001**
	4W	-6.31 (-6.90, -5.71; **< 0.001**)	-6.57 (-7.25, -5.89; **< 0.001**)	-8.14 (-8.84, -7.44; **< 0.001**)	0.02 (-0.88, 0.91;0.973)	1.82 (0.97, 2.66; **< 0.001**)	1.8 (0.84, 2.76; **< 0.001**)	
	8W	-6.8 (-7.31, -6.29; **< 0.001**)	-8.06 (-8.77, -7.35; **< 0.001**)	-9.42 (-10.06, -8.77; **< 0.001**)	1.02 (0.13, 1.90; **0.025**)	2.6 (1.83, 3.37; **< 0.001**)	1.58 (0.66, 2.51; **< 0.001**)	
	12W	-8.55 (-9.09, -8.02; **<0.001**)	-10 (-10.81, -9.19; **<0.001**)	-12.25 (-12.95,-11.5; **<0.001**)	1.2 (0.31, 2.09; **0.008**)	3.68 (2.95, 4.41; **<0.001**)	2.48 (1.50, 3.46; **< 0.001**)	

Data are shown as mean ± standard deviation. E/D group as control group; ESC group:escitalopram group; Comb group: Combined group; HAMD, Hamilton Depression Rating Scale; HAMA, Hamilton Anxiety Scale; The changes were processed by generalized estimation equations (GEEs) analysis. Intervention effects on these variables were examined by GEEs, with time (number of weeks from baseline), group, and group-by-time interaction as covariates. A significant group-by-time interaction indicated a significant difference for a given variable between interventions over time. Pairwise treatment comparisons were performed by linear contrast with Bonferroni correction to adjust for multiple comparisons. P values in boldface indicate significance after Bonferroni correction.

In the between-group comparison, the Comb group demonstrated consistently lower scores on the HAMA scales across all time points (weeks 2, 4, 8, and 12) when compared with the other two groups. At weeks 4, 8, and 12, the HAMD score was significantly lower in the ESC group than in the E/D group, while that of the Comb group was markedly lower than that of the other two groups; and at weeks 8 and 12, the HAMA score was significantly lower in the ESC group than in the E/D group.

### 3.3 Secondary endpoints

Changes in the secondary endpoint indicators are shown in **Tables 3**, **4** and **Figure 2**. Compared with the baseline data, all three groups reported a gradual decrease in PHQ-15, PHQ-9, GAD-7 scores (P<0.001 for all), and a gradual increase in serum E2 and 5-HT (P<0.001 for both), with the most significant change in the Comb group.

**Table 3:** Change from baseline in PHQ-15, PHQ-9, and GAD-7 scores.

Item	Time	Changes from Baseline (95%CI; P)			Inter-group difference (95%CI; P)			Group-by-timeInter-actionEffect
		E/D group	ESC group	Comb group	E/D groupvs ESC group	E/D groupvs*Comb group*	ESC groupvs Comb group	
PHQ-15 Score	2W	-1.80 (-2.19, -1.41; **< 0.001**)	-1.88 (-2.22, -1.53; **< 0.001**)	-2.58 (-2.91, -2.25; **< 0.001**)	-0.22 (-0.83, 0.40; 0.493)	0.80 (0.21, 1.39; **0.008**)	1.02 (0.48, 1.55; **< 0.001**)	**< 0.001**
	4W	-2.66 (-3.08, -2.24; **< 0.001**)	-2.91 (-3.35, -2.47; **< 0.001**)	-3.58 (-3.96, -3.21; **< 0.001**)	-0.05 (-0.69, 0.60; 0.888)	0.94 (0.32, 1.55; **0.003**)	0.98 (0.40, 1.57; **< 0.001**)	
	8W	-3.91 (-4.29, -2.52; **< 0.001**)	-4.26 (-4.72, -3.80; **< 0.001**)	-4.85 (-5.33, -4.37; **< 0.001**)	0.06 (-0.50, 0.62; 0.829)	0.95 (0.43, 1.48; **< 0.001**)	0.89 (0.38, 1.41; **< 0.001**)	
	12W	-4.23 (-4.63, -3.84; **< 0.001**)	-4.71 (-5.15, -4.27; **< 0.001**)	-5.63 (-6.05, -5.22; **< 0.001**)	0.18 (-0.34, 0.71; 0.488)	1.42 (1.01, 1.82; **< 0.001**)	1.23 (0.76, 1.70; **< 0.001**)	
GAD-7 Score	2W	-1.11 (-1.44, -0.78; **< 0.001**)	-1.37 (-1.69, -1.05; **< 0.001**)	-1.95 (-2.36, -1.54; **< 0.001**)	-0.06 (-0.59, 0.47;0.819)	0.60 (0.03, 1.17; **0.039**)	0.66 (0.10, 1.22; **0.020**)	**< 0.001**
	4W	-2.17 (-2.55, -1.78; **< 0.001**)	-2.58 (-2.96, -2.21; **< 0.001**)	-3.54 (-3.99, -3.08; **< 0.001**)	0.09 (-0.39, 0.57;0.706)	1.12 (0.67, 1.58; **< 0.001**)	1.03 (0.54, 1.52; **< 0.001**)	
	8W	-3.08 (-3.44, -2.71; **< 0.001**)	-3.57 (-3.97, -3.17; **< 0.001**)	-4.34 (-4.76, -3.92; **< 0.001**)	0.17 (-0.35, 0.69;0.526)	1.02 (0.48, 1.56; **< 0.001**)	0.85 (0.35, 1.34; **< 0.001**)	
	12W	-3.40 (-3.81, -2.99; **< 0.001**)	-4.31 (-4.83, -3.78;< 0.001)	-5.71 (-6.18, -5.24; **< 0.001**)	0.58 (0.02, 1.15; **0.041**)	2.06 (1.52, 2.60; **< 0.001**)	1.48 (0.91, 2.05; **< 0.001**)	
PHQ-9 Score	2W	-1.00 (-1.40, -0.60; **< 0.001**)	-1.78 (-2.22, -1.35; **< 0.001**)	-2.37 (-2.89, -1.85; **< 0.001**)	0.52 (-0.33, 1.38;0.232)	1.71 (0.63, 2.78; **0.002**)	1.18 (0.13, 2.24; **0.028**)	**< 0.001**
	4W	-1.43 (-1.85, -1.02; **< 0.001**)	-2.88 (-3.37, -2.39; **< 0.001**)	-3.45 (-3.91, -2.98; **< 0.001**)	1.18 (0.26, 2.11; **0.012**)	2.35 (1.40, 3.31; **< 0.001**)	1.17 (0.21, 2.13; **0.017**)	
	8W	-2.68 (-3.15, -2.20; **< 0.001**)	-3.71 (-4.21, -3.20; **< 0.001**)	-4.26 (-4.77, -3.75; **< 0.001**)	0.77 (-0.01, 1.55;0.054)	1.92 (1.11, 2.73; **< 0.001**)	1.15 (0.32, 1.99; **0.007**)	
	12W	-3.06 (-3.51, -2.61; **< 0.001**)	-4.55 (-5.10, -4.01; **< 0.001**)	-5.60 (-6.22, -4.98; **< 0.001**)	1.23 (0.38, 2.08; **0.005**)	2.88 (2.02, 3.73; **< 0.001**)	1.65 (0.84, 2.46; **< 0.001**)	

Data are shown as mean ± standard deviation. E/D group as control group; ESC group:escitalopram group; Comb group: Combined group; PHQ-15, Patient Healthy Questionnaire-15; GAD-7, Generalized Anxiety Disorder-7; PHQ-9, Patient Health Questionnaire-9; The changes were processed by generalized estimation equations (GEEs) analysis. Intervention effects on these variables were examined by GEEs, with time (number of weeks from baseline), group, and group-by-time interaction as covariates. A significant group-by-time interaction indicated a significant difference for a given variable between interventions over time. Pairwise treatment comparisons were performed by linear contrast with Bonferroni correction to adjust for multiple comparisons. P values in boldface indicate significance after Bonferroni correction.

**Table 4:** Change from baseline in serum 5-HT and E2 levels at secondary endpoints.

Item	Time	Changes from Baseline (95%CI; P)			Inter-group difference (95%CI; P)			Group-by-timeInter-actionEffect
		E/D group	ESC group	Comb group	E/D groupvs ESC group	E/D groupvs*Comb group*	ESC groupvs Comb group	
Serum E2, pg/ml	2W	6.74 (6.25, 7.22; **< 0.001**)	0.84 (0.48, 1.20; **< 0.001**)	6.75 (6.17, 7.32; **< 0.001**)	4.98 (3.72, 6.23; **< 0.001**)	-0.18 (-1.38, 1.01;0.763)	-5.16 (-6.42, -3.90; **< 0.001**)	**< 0.001**
	4W	8.44 (7.83, 9.06; **< 0.001**)	2.97 (2.38, 3.57; **< 0.001**)	9.44 (8.70, 10.18; **<0.001**)	4.55 (3.37, 5.74; **< 0.001**)	-1.17 (-2.23, -0.11; **0.030**)	-5.72 (-6.82, -4.62; **< 0.001**)	
	8W	12.03 (11.31, 12.75; **< 0.001**)	3.95 (3.20, 4.70; **< 0.001**)	12.31 (11.37, 13.25; **< 0.001**)	7.16 (6.06, 8.26; **< 0.001**)	-0.45 (-1.46, 0.55;0.375)	-7.61 (-8.60, -6.62; **< 0.001**)	
	12W	6.74 (14.16, 13.32; **< 0.001**)	5.18 (4.43, 5.93; **< 0.001**)	14.70 (13.75, 15.65; **< 0.001**)	8.07 (6.92, 9.21; **< 0.001**)	-0.71 (-1.76, 0.35;0.189)	-8.78 (-9.73, -7.82; **< 0.001**)	
Serum 5-HT, ng/ml	2W	20.11 (12.33, 27.90; **< 0.001**)	26.59 (18.25, 34.93; **< 0.001**)	33.56 (26.13, 40.99; **< 0.001**)	-2.74 (-12.75, 7.26;0.591)	-8.75 (-18.44, 0.94;0.077)	-6.01 (-14.96, 2.95;0.189)	**< 0.001**
	4W	17.50 (10.76, 24.25; **< 0.001**)	25.51 (17.25, 33.77; **< 0.001**)	37.99 (30.65, 45.33; **< 0.001**)	-4.27 (-12.72, 4.18;0.322)	-15.79 (-23.86, -7.71; **< 0.001**)	-11.51 (-19.47, -3.56; **0.005**)	
	8W	23.68 (16.69, 30.67; **< 0.001**)	33.90 (25.52, 42.28; **< 0.001**)	42.32 (35.07, 49.57; **< 0.001**)	-6.48 (-15.37, 2.41;0.153)	-13.94 (-23.02, -4.85; **0.003**)	-7.45 (-16.20, 1.29;0.095)	
	12W	22.31 (15.74, 28.88; **< 0.001**)	39.08 (30.89, 47.28; **< 0.001**)	50.05 (43.13, 56.98; **< 0.001**)	-13.04 (-21.41, -4.66; **0.002**)	-23.04 (-30.59, -15.50; **< 0.001**)	-10.00 (-18.04, -1.97; **0.015**)	

Data are shown as mean ± standard deviation. E/D group as control group; ESC group:escitalopram group; Comb group: Combined group; 5-HT, 5-hydroxytryptamine; E2, Estradiol. The changes were processed by generalized estimation equations (GEEs) analysis. Intervention effects on these variables were examined by GEEs, with time (number of weeks from baseline), group, and group-by-time interaction as covariates. A significant group-by-time interaction indicated a significant difference for a given variable between interventions over time. Pairwise treatment comparisons were performed by linear contrast with Bonferroni correction to adjust for multiple comparisons. P values in boldface indicate significance after Bonferroni correction.

In the between-group comparison, the Comb group demonstrated consistently lower scores on the PHQ-15, GAD-7, and PHQ-9 scales across all time points (weeks 2, 4, 8, and 12) when compared with the other two groups. At week 12, the GAD-7 score was lower in the ESC group than in the E/D group (P = 0.041); at weeks 4 and 12, the PHQ-9 score was significantly lower in the ESC group than in the E/D group.

Meanwhile, at weeks 2, 4, 8, and 12, the E2 level was significantly higher in the Comb and E/D groups than in the ESC group; at week 4, the E2 level was significantly higher in the Comb group than in the E/D group; at weeks 4, 8, and 12, the 5-HT level was significantly higher in the Comb group than in the E/D group; and at week 4 and 12, the 5-HT level was significantly higher in the Comb group than in the ESC group.

### 3.4 Mediation analysis

**Figure 3:**
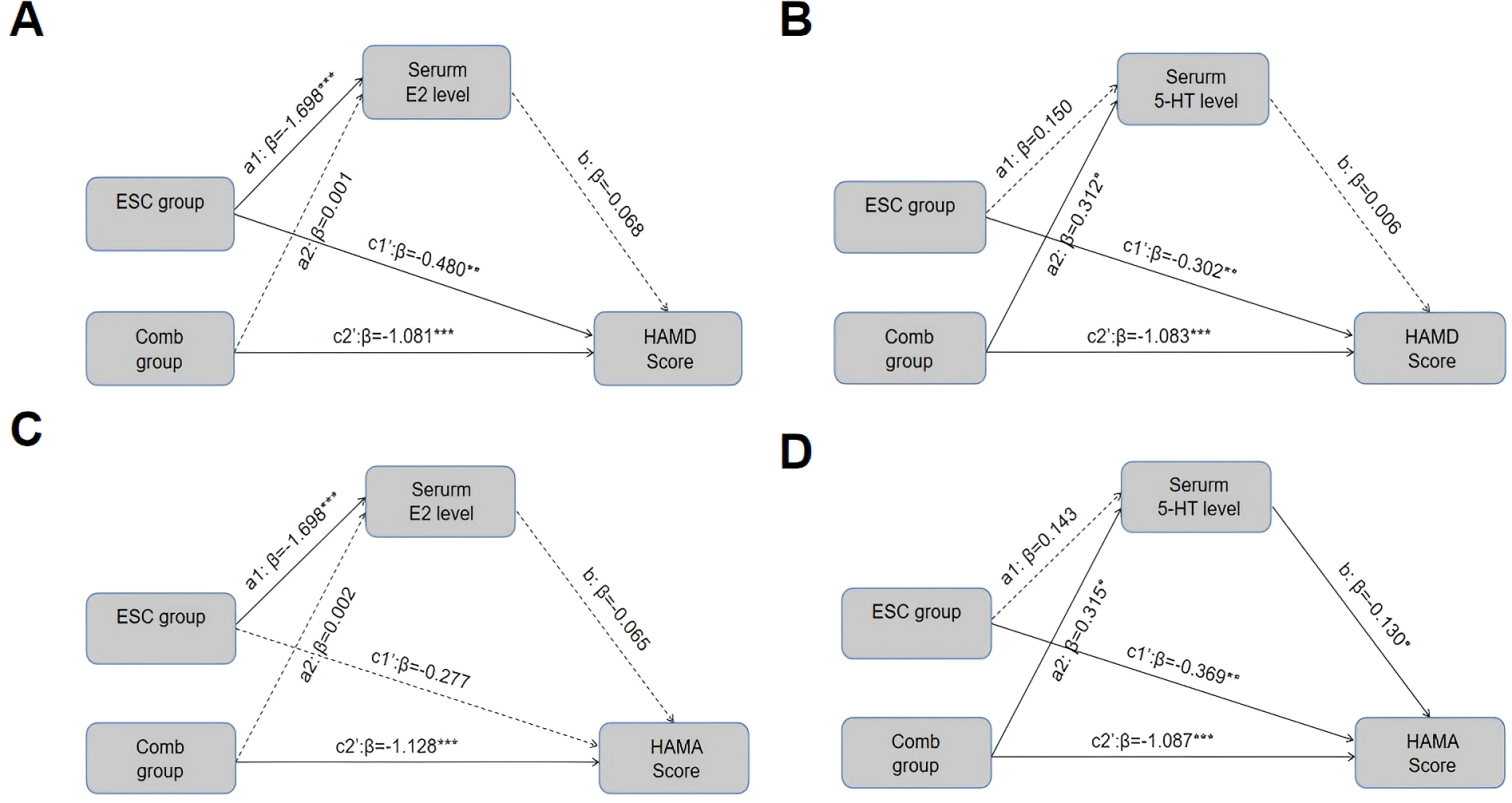
Models tested in the mediation analysis. **(A)** Mediating effect of serum E2 between treatment subgroups and improvement in HAMD scores; **(B)** Mediating effect of serum 5-HT between treatment subgroups and improvement in HAMD scores; **(C)** Mediating effect of serum E2 between treatment subgroups and improvement in HAMA scores; **(D)** Mediating effect of serum 5-HT between treatment subgroups and improvement in HAMA scores; dashed lines indicate paths that are not significant. a1, a2, and b denote direct pathways; c1’ and c2’ indicate indirect pathways [group → serum E2/serum 5-HT → HAMD/HAMA]; β is the standardized regression coefficients after the adjustment for covariates (age, years of education, BMI, and baseline levels of the corresponding M,Y). *P < 0.05, **P < 0.01, ***P < 0.001. HAMD, Hamilton Depression Rating Scale; HAMA, Hamilton Anxiety Scale; 5-HT, 5-hydroxytryptamine; E2, Estradiol.

Mediation analyses were performed to determine whether the improvement in perimenopausal anxiety and perimenopausal depression by the 12-week combination therapy was mediated by the fluctuation in E2 and 5-HT levels in these patients (**Figure 3**, **Supplementary Tables 1**, **2**). The results revealed no mediating effect of the increased E2 and 5-HT levels on the beneficial outcome of the combined therapy.

### 3.5 Correlation analysis

**Figure 4:**
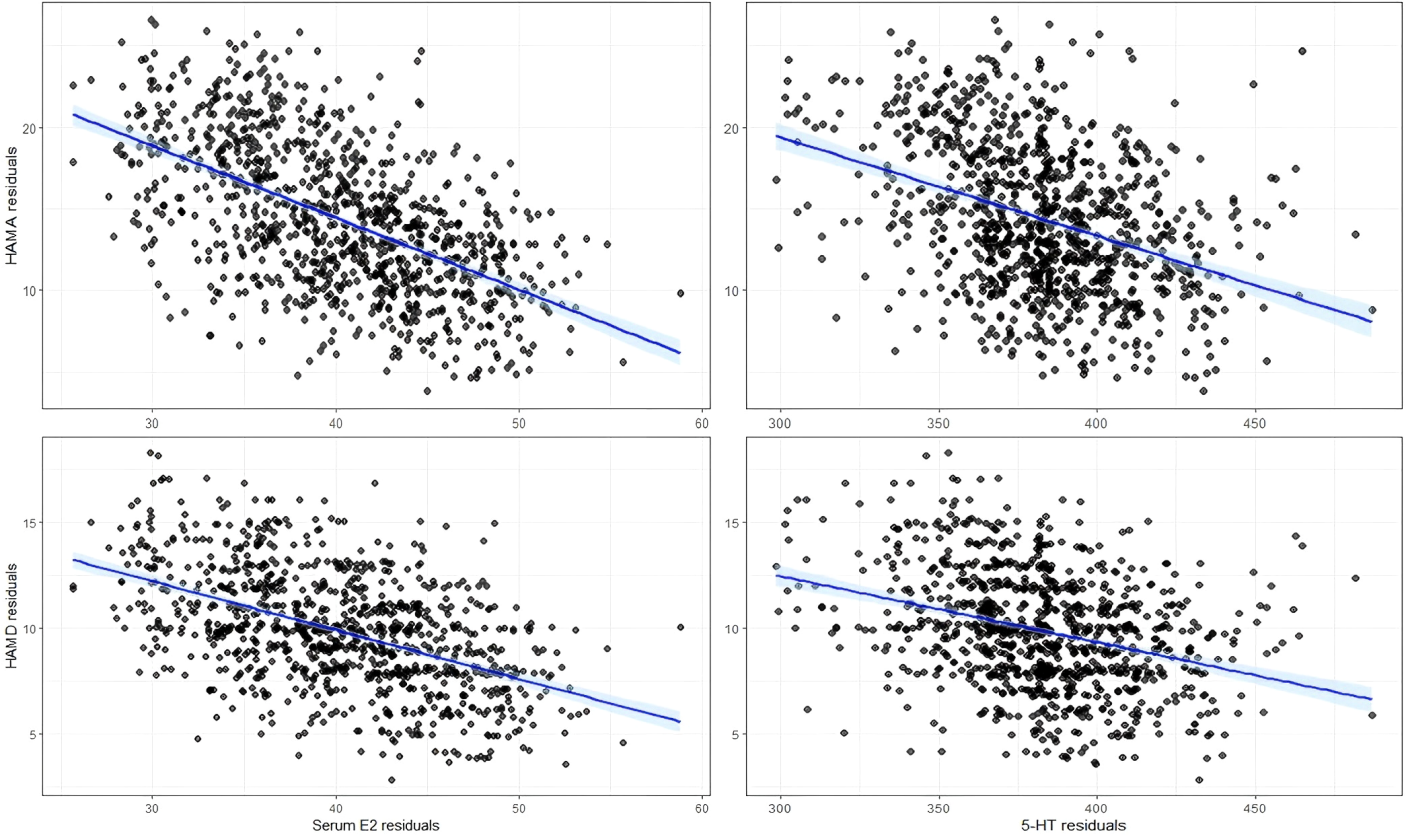
Correlation between serum E2 and 5-HT levels and HAMA and HAMD scores. HAMD, Hamilton Depression Rating Scale; HAMA, Hamilton Anxiety Scale; 5-HT, 5-hydroxytryptamine; E2, Estradiol.

The correlations of blood indices with HAMD and HAMA scores across the three groups at baseline and post-treatment weeks 2, 4, 8, and 12 are detailed in **Figure 4** and **Supplementary Table 3**. As shown, the level of both serum E2 and 5-HT was moderately negatively correlated with HAMA and HAMD scores.

### 3.5 AEs

All treatments were well-tolerated and safe. As detailed in **Supplementary Table 4**, no serious adverse reactions were observed, and no significant differences were found in the total incidence of adverse reactions or in pre-versus post-treatment laboratory parameters (blood routine, liver/kidney function, electrocardiograms) among the groups.

## 4 Discussion

In this study, we found that estradiol/dydrogesterone combined with escitalopram more effectively ameliorated anxiety and depression symptoms in perimenopausal women when compared with either escitalopram or estradiol/dydrogesterone. After 12 weeks of treatment, all groups showed a decrease in HAMA, HAMD, PHQ-15, PHQ-9, and GAD-7 scores, and an increase in serum E2 and 5-HT levels, with the most significant changes observed in the Comb group. The incidence of adverse events was comparable between the three groups, indicating no additional safety risks from the combination therapy. Moreover, the levels of both serum E2 and 5-HT were moderately negatively correlated with HAMA and HAMD scores. These findings may provide new clinical insights into the treatment of perimenopausal anxiety and depression.

Previous studies have demonstrated that either estrogen therapy or escitalopram can improve perimenopausal depressive symptoms (Kulkarni et al., 2018; Lozza-Fiacco et al., 2022; Freeman et al., 2006). A study of symptomatic perimenopausal and postmenopausal women has found that escitalopram is more effective in treating depression than estrogen-progestin therapy (Soares et al., 2006). Consistently, the current study found that at week 12, the scores of the anxiety-depression scale were significantly lower in the ESC group than in the E/D group. Moreover, estrogen has been found to enhance the efficacy of antidepressants in the treatment of perimenopausal depression (Kornstein et al., 2013; Morgan et al., 2005). Similarly, the results of our study revealed that the combined therapy greatly mitigated anxiety and depression in perimenopausal women. These findings suggest that estrogenic agents may serve as a promising candidate for the treatment of perimenopausal anxiety and depression.

To date, the mechanisms underlying perimenopausal anxiety and depression. await elucidation. Some studies have found that the decrease of estrogen may play an essential role in the development of perimenopausal depression and anxiety (Gordon et al., 2019; Joffe et al., 2020; Schmidt et al., 2015; Lozza-Fiacco et al., 2022). Other studies have suggested that the fluctuation of perimenopausal estradiol level may lead to the dysregulation of neurotransmitters (e.g., dopamine, serotonin, and norepinephrine) and ultimately contribute to perimenopausal anxiety and depression (Soares, 2014). Still others have indicated that the fluctuation of estradiol and luteinizing hormone may induce the fluctuation of allopregnanolone, which acts on the g-aminobutyric acid A receptor and affects the hypothalamic-pituitary-adrenal axis, thus in turn impacting mood (Gordon et al., 2021). Meanwhile, estrogen has been demonstrated to be associated with the synthesis and reuptake of 5-HT and the number of its receptors, which can modulate the mood-related 5-HT pathway (Sánchez et al., 2013; Bethea et al., 2000; Gundlah et al., 2002; Zeng et al., 2025). In a similar line, the current study showed that the level of serum E2 and 5-HT was elevated in all three groups after the respective treatment, with more pronounced effects observed in the Comb group. Altogether, these results evidence that both estrogen and 5-HT may play a role in the development of perimenopausal anxiety and depression and that the combination therapy may act on both neuroendocrine and monoaminergic systems. However, the exact mechanism needs further explorations.

After confirming the clinical benefits of the combination therapy, we further explored the mechanisms underlying the potential mediation of serum E2 and 5-HT. Of interest, although the elevation of serum E2 and 5-HT levels was significantly associated with the decrease of HAMA/HAMD scores, no significant mediation effect was observed for either of them. This is inconsistent with the previous findings that suggest E2 fluctuation is associated with mood severity (Schmidt et al., 2015; Lozza-Fiacco et al., 2022; Gordon et al., 2021).

One plausible explanation for this inconsistency may lie in that escitalopram and estradiol/dydrogesterone may ameliorate perimenopausal anxiety and depressive symptoms through other pathways. Specifically, although it has been shown that serum E2 levels are associated with perimenopausal mood, it may act indirectly through other pathways rather than directly on mood centers. For example, a longitudinal study examining hormone levels and depression in perimenopausal women suggests that perimenopausal emotional problems are more likely to result indirectly from the vasomotor symptoms (VMS) induced by reduced estrogen (Avis et al., 2001). Several other studies have found a specific link between anxiety and VMS (Bromberger et al., 2013; Freeman and Sammel, 2016). Still another study indicates that more profound mechanism may lie in the ‘moderating’ rather than ‘mediating’ role of serum E2, whereby the fluctuations in perimenopausal estradiol may elevate the body’s sensitivity to stress through the GABA-hypothalamic-pituitary-adrenal axis system, thus making the perimenopausal women more susceptible to depressive tendencies when under the psychosocial stress (Gordon et al., 2019). Finally, escitalopram and estradiol/dydrogesterone treatment may also improve anxiety and depression symptoms by improving other hormone levels such as norepinephrine, as well as Brain-Derived Neurotrophic Factor levels (Jiang et al., 2017; Wen et al., 2025). In conclusion, the mechanisms by which escitalopram and estradiol/dydrogesterone ameliorate perimenopausal anxiety and depressive symptoms are complex and involve complex moderating effects on social, psychological, and physiological aspects, which need to be followed up with further studies.

In the final analysis, in the current study, the combined treatment of estradiol/dydrogesterone with escitalopram offers a more efficient and safety-controlled option for perimenopausal anxiety and depression. The combination treatment strategy may be particularly beneficial for patients who do not respond well to single SSRI treatment. In addition, no significant differences in the incidence of AEs were observed between the Comb group and the other two groups, suggesting the safety of the combination therapy.

Some limitations remain in this study. First, this is a small-sample study and mediation analyses usually require a large sample size. Therefore, our sample size may not be sufficient to detect small but meaningful mediating effects. Second, this is a short-term follow-up study. The 12-week duration may not be sufficient to observe the long-term efficacy, stability, and potential adverse effects. Third, some potentially important variables were not included in the analysis, including other hormone levels, social factors, metabolic indicators (e.g., body weight and body composition), and lifestyle factors (e.g., physical activity, diet, and sleep). Given that weight gain is a significant metabolic change during the perimenopausal period and may be associated with SSRI treatment, and that decreased physical activity is closely related to depression and anxiety, the lack of systematic assessment of these variables limits our comprehensive understanding of the overall effects and potential confounding influences on the interventions. Finally, as this study included perimenopausal women with mild-to-moderate anxiety disorders and depressive disorders the efficacy of the combined treatment for those without anxiety disorders and those with major anxiety disorders and major depressive disorders remains unknown and needs to be further investigated.

In summary, this study demonstrates that the combination of estradiol/dydrogesterone and escitalopram can yield significant efficacy for perimenopausal anxiety and depression with a favorable safety profile over a 12-week study period. Although serum E2 and 5-HT levels are associated with clinical improvement, they do not appear to be the primary mediators of the unique benefits of the combination therapy.

## Data Availability

The data that support the findings of this study are available from the corresponding author. The data are not publicly available due to privacy or ethical restrictions.
