# Supplementary material for: Efficacy and safety of estradiol/dydrogesterone combined with escitalopram in the treatment of anxiety and depression in perimenopausal women: a randomized controlled trial
Source: Front Physiol. 2026 Apr 2;17:1728736. doi: 10.3389/fphys.2026.1728736 (PMC13082949; doi:10.3389/fphys.2026.1728736)
Supplement: Supplementary file 1 [file DataSheet1.docx]

**ELECTRONIC SUPPLEMENTARY MATERIAL**

**Efficacy and safety of dydrogesterone/estradiol combined with escitalopram in the treatment of anxiety and depression in perimenopausal women: A randomized controlled trial**

**SUPPLEMENTARY METHODS**

**Study Design**

*Sample size.* This study was an exploratory trial, with no data reference. Hence, the sample size was not estimated.

*Recruitment.* All interested patients were given a patient information sheet. If they agreed to participate, a signed informed consent was obtained and their medical data were accessed.

*Screening visit.* All participants were screened with the Structured Clinical Interview for DSM-5 Disorders (SCID-5) to confirm a diagnosis of anxiety or depressive disorder (First et al., 2015). In the study, HAMD-17, HAMA-14, GAD-7, and PHQ-9 scales were used to assess the emotional status of patients in each group and the PHQ-15 was employed to evaluate somatic symptoms. Meanwhile, blood samples were collected from the participants for the measurements of serum E2 and 5-HT levels, full blood count, and parameters of liver and kidney function. Additionally, the vital signs, height, and weight of the participant were recorded.

*Baseline visit.* After the data screening, qualified patients returned for baseline follow-up. They were randomly assigned to the D/E group or the escitalopram group or the comb group. Each group underwent testing of blood monoamine neurotransmitters (serum 5-HT and E2) and clinical assessment of the HAMD-17, HAMA-14, GAD-7, PHQ-9, PHQ-15 scales.

*Randomization.* Patients were numbered according to the enrollment sequence and randomly grouped at a 1:1:1 allocation ratio according to the order of random numbers generated with SPSS 17.0 software (SPSS Inc., Chicago, IL, USA). An independent researcher used the algorithm to perform rater masking. The randomized information of each eligible patient was sealed in an opaque envelope that corresponded to the patient enrollment number. Participants were randomized into three 12-week treatment regimens: escitalopram + placebo (ESC group), dydrogesterone/estradiol + placebo (D/E group), and dydrogesterone/estradiol plus escitalopram (Comb group). The ESC group received escitalopram (Bailot, Sichuan Kelun Pharmaceutical Co., Ltd.) at a dosage of 10 mg once daily. In case of adverse events (AEs), the dose was reduced to 5 mg once daily for 3 days and increased to 10 mg for maintenance therapy if the AEs were relieved. The placebo was an analogue to dydrogesterone/estradiol and administered in the same dosage regimen of dydrogesterone/estradiol. The D/E group received dydrogesterone/estradiol 1/10 (Solvay Pharmaceutical Co., Ltd.), which consists of a white tablet (estradiol 1mg) and a gray tablet (estradiol 1mg + dydrogesterone 10mg). Specifically, an initial 28-day course of one tablet daily involved the white tablet taken for the first 14 days and the gray tablet for the next 14 days; and a second course started from day 29. If the discomfort associated with estrogen insufficiency did not improve, the patients were given dydrogesterone/estradiol 2/10 (Solvay Pharmaceuticals Ltd.), composed of a brick red tablet (estradiol 2 mg) and a yellow tablet (estradiol 2 mg + dydrogesterone 10 mg), following the same dosage scheme. The placebo was an escitalopram analogue, given at a dosage of 10 mg once daily. The Comb group received dydrogesterone/estradiol and escitalopram simultaneously according to the above schema.The dosage form, color and taste of the placebo were consistent with the actual use of escitalopram or dydrogesterone/estradiol tablets. The participants, clinicians, and outcome assessors were unaware of subgroups. All researchers involved in drug administration received uniform training to avoid inadvertent disclosure of grouping information.

Before treatment, all enrolled patients received a 1-week drug washout (2 weeks for those already taking a monoamine oxidase inhibitor).

During the treatments, contraindicated drugs were as follows: triptans, antipsychotics, catecholamines, glucocorticosteroids, monoamine oxidase inhibitors and β-receptor blockers. The use of following drugs was allowed: antiplatelet agents, anticoagulants, B vitamins, angiotensin-converting enzyme inhibitors, angiotensin receptor blockers, and calcium channel antagonists.

*Follow-up visit*. Participants were assessed at weeks 2, 4, 8, and 12 after the treatment, within 3 days of the end of the session. Participants underwent a clinical assessment with the same measurements used at baseline follow-up, and blood was taken for analyses of monoamine neurotransmitter and hormone levels.

All assessments were conducted by two neurologists (one senior attending physician and one associate chief physician), who were trained in scale consistency and masked to the grouping and medication of patients.

**Assessment of Emotional and Somatic Symptoms**

*Patient Healthy Questionnaire-15 Scale*: It mainly assesses the severity of somatic symptoms. It consists of 15 items, covering common somatic symptoms such as fatigue, sleep problems, dizziness, and headache. The scale uses a 3-point scoring system (0-2 points), with a total score ranging from 0 to 30. A higher score indicates severer somatic symptoms. Specifically, a total score of 0-4 points means no somatic symptoms; 5-9 points indicates mild somatic symptoms; 10-14 points denotes moderate somatic symptoms; and a score of ≥15 points represents severe somatic symptoms.

*Patient Health Questionnaire-9 Scale:* The scale was adopted for the assessment of depressive symptoms. It consists of 9 items, corresponding to the 9 core symptom criteria for depression in the Diagnostic and Statistical Manual of Mental Disorders diagnostic system. The scale uses a 4-point scoring system (0-3 points), with a total score ranging from 0 to 27. Specifically, a total score of 0-4 points indicates no depression; 5-9 points indicates mild depression; 10-14 points moderate depression; 15-19 points moderately severe depression; and 20-27 points severe depression.

*Generalized Anxiety Disorder-7 Scale：*The scale was adopted for the assessment of anxiety symptom severity. It includes 7 core anxiety symptoms (such as excessive worry, restlessness), with a total score ranging from 0 to 21. Specifically, a total score of 0-4 points indicates no anxiety; 5-9 points indicates mild anxiety; 10-14 points denotes moderate anxiety; and a score of ≥15 points indicates severe anxiety.

*The 17-item Hamilton Depression Rating Scale:* The 17-item Hamilton Depression Rating Scale (HAMD-17) was used to assess the severity of depression in patients. Each item of the scale is scored on a range of 0 to 4 points, with higher scores indicating severer symptoms. HAMD-17 boasts a good reliability, validity, and stability, and can be used for screening depressive symptoms in primary care populations. The scale classifies depressive symptoms into four grades: no depression (0-7 points), mild depression (8-16 points), moderate depression (17-23 points), and severe depression (≥24 points).

*The 14-item Hamilton Anxiety Rating Scale (HAMA-14):* It was used to assess the anxiety symptoms of enrolled patients. This scale has a good reliability and validity, and is widely used to measure the anxiety level of patients with depressive symptoms. The HAMA-14 Scale includes 14 assessment items, covering aspects such as tension, insomnia, somatic symptoms, and gastrointestinal symptoms. Each item is scored on a scale of 0 to 4, with higher scores indicating severer symptoms. A total score below 7 points indicates no anxiety; a total score of 14 points or above clearly suggests the presence of anxiety symptoms; a total score of 21 points or above indicates significant anxiety; and a total score of 29 points or above indicates severe anxiety.

**Blood Sample Detection**

*Collection of blood samples:* All participants had blood samples collected before the treatment and at weeks 2, 4, 8 and 12 after the treatment. Three days before the blood collection, patients were required to avoid foods that affect the secretion of monoamines and cholinergics, and to observe fasting at 22:00 on the evening of the day before the blood collection. Blood collection was performed from 08:00 to 10:00 am after a 30-minute peaceful resting. Venous blood was collected from the elbow vein into ethylenediaminetetraacetic acid (EDTA) anticoagulant tubes (1 tube) and procoagulant tubes (2 tubes), followed by platelet and serum extraction.

*Sample handling and detection:* Platelets were extracted with a human peripheral platelet separation kit (Solarbio, Beijing, China) and serum was extracted by a low-speed benchtop centrifuge (Shanghai Anting Scientific Instrument Factory, China, No. type: TDL-50B). Platelets and serum were frozen at -80 °C to avoid repeated freeze-thaw and the storage time was not more than 2 months. Enzyme-linked immunosorbent assay (ELISA) was adopted to measure serum E2 and 5-HT levels.

**Reference**

First, M., Williams, J., Karg, R., & Spitzer, R. (2015). Structured clinical interview for DSM-5 disorders, Research Version (SCID-5-RV). Arlington, VA: American Psychiatric Publishing.

**SUPPLEMENTARY TABLES**

| **Item** | ***β* (95% CI)** | **Std *β*** | ***P*** |
| --- | --- | --- | --- |
| **Path** |  |  |  |
| X₁ → M₁ | -5.819(-6.520, -5.117) | -1.698 | **<0.001** |
| X₂ → M₁ | 0.005(-0.693, 0.702) | 0.001 | 0.990 |
| M₁ → Y | -0.068(-0.171, 0.035) | -0.106 | 0.193 |
| X₁ → M₂ | 4.926(-4.711, 14.563) | 0.150 | 0.315 |
| X₂ → M₂ | 10.269(0.685, 19.854) | 0.312 | **0.036** |
| M₂ → Y | 0.000(-0.007, 0.008) | 0.006 | 0.918 |
| **Relative total effect** |  |  |  |
| X₁ → Y | -0.667(-1.166, -0.167) | -0.302 | **0.009** |
| X₂ → Y | -2.393(-2.890, 1.896) | -1.081 | **<0.001** |
| **Relative direct effect** |  |  |  |
| X₁ → Y (Controlling for M₁) | -1.063(-1.843, - 0.284) | -0.480 | **0.008** |
| X₂ → Y (Controlling for M₁) | -2.393(-2.889, -1.897) | -1.081 | **<0.001** |
| X₁ → Y (Controlling for M₂) | -0.669(-1.171, -0.166) | -0.302 | **0.009** |
| X₂ → Y (Controlling for M₂) | -2.397(-2.901, 1.893) | -1.083 | **<0.001** |
| **Relative indirect effect** |  |  |  |
| X₁ → M₁ → Y | 0.397(-0.151, 1.016) | 0.179 | / |
| X₂ → M₁ → Y | -0.000(-0.078, 0.059) | -0.000 | / |
| X₁ → M₂ → Y | 0.002(-0.060, 0.061) | 0.001 | / |
| X₂ → M₂ → Y | 0.004(-0.094, 0.094) | 0.002 | / |

**Table S1 Mediating effect of E2 and 5-HT on HAMD in the ESC and Comb groups.**

D/E group as control group; ESC group：escitalopram group；Comb group: Combined group；X₁，ESC group；X₂，Comb group；M₁, E2; M₂, 5-HT；Y, HAMD; Std *β*, standardized ***β***. Standardized ***β*** indicates the standardized regression coefficient and is adjusted for covariates (age, education years, BMI, and the corresponding baseline levels of M and Y). *P* values in boldface indicate significance.

**Table S2 Mediating effect of E2 and 5-HT on HAMA in the ESC and Comb groups.**

| **Item** | ***β* (95% CI)** | **Std *β*** | ***P*** |
| --- | --- | --- | --- |
| **Path** |  |  |  |
| X₁ → M₁ | -5.819(-6.520, 5.118) | -1.698 | **<0.001** |
| X₂ → M₁ | 0.005(-0.692, 0.703) | 0.002 | 0.988 |
| M₁ → Y | 0.061(-0.104, 0.227) | 0.065 | 0.466 |
| X₁ → M₂ | 4.697(-4.962, 14.356) | 0.143 | 0.339 |
| X₂ → M₂ | 10.360(0.749, 19.971) | 0.315 | **0.035** |
| M₂ → Y | -0.013(-0.025, -0.001) | -0.130 | **0.035** |
| **Relative total effect** |  |  |  |
| X₁ → Y | -1.255(-2.054, -0.456) | -0.387 | **0.002** |
| X₂ → Y | -3.654,(-4.450, -2.859) | -1.128 | **<0.001** |
| **Relative direct effect** |  |  |  |
| X₁ → Y（Controlling for M₁） | -0.899(-2.150, 0.353) | -0.277 | 0.158 |
| X₂ → Y (Controlling for M₁) | -3.654(-4.451, -2.858) | -1.128 | **<0.001** |
| X₁ → Y (Controlling for M₂) | -1.195(-1.989, -0.401) | -0.369 | **0.003** |
| X₂ → Y (Controlling for M₂) | -3.522(-4.319, -2.724) | -1.087 | **<0.001** |
| **Relative indirect effect** |  |  |  |
| X₁ → M₁ → Y | -0.359(-1.242, 0.546) | -0.110 | / |
| X₂ → M₁ → Y | 0.000(-0.075, 0.087) | 0.000 | / |
| X₁ → M₂ → Y | -0.060(-0.250, 0.067) | -0.019 | / |
| X₂ → M₂ → Y | -0.133(-0.360, 0.003) | -0.041 | / |

D/E group as control group; ESC group：escitalopram group；Comb group: Combined group；X₁，ESC group；X₂，Comb group；M₁, E2；M₂, 5-HT；Y, HAMA; Std *β*, standardized ***β***. Standardized ***β*** means the standardized regression coefficient and is adjusted for covariates (age, education years, BMI, and the corresponding baseline levels of M and Y). *P* values in boldface indicate significance.

**Table S3 Pearson's partial correlation coefficient (r) between clinical efficacy and peripheral blood indices.^a^**

| Item | HAMD | |  | HAMA | |
| --- | --- | --- | --- | --- | --- |
|  | r | P value |  | r | P value |
| Serum E2 | -0.483 | ＜0.001 |  | -0.551 | ＜0.001 |
| Serum 5-HT | -0.329 | ＜0.001 |  | -0.378 | ＜0.001 |

Data are shown as correlation coefficients. PHQ-15, Patient Healthy Questionnaire-15; HAMD, Hamilton Depression Rating Scale; HAMA, Hamilton Anxiety Scale; 5-HT, 5-hydroxytryptamine; E2, Estradiol

^a^ Partial correlation analyses were adjusted for age, years of education, and BMI level.

**Table S4 Comparison of adverse event rates.**

| **Time** | **D/E group**  **(n=65)** | **ESC group**  **(n=65)** | **Comb group (n=65)** | **χ² value** | **^#^*P value*** |
| --- | --- | --- | --- | --- | --- |
| 0-2W | 4（6.2%） | 5（7.7%） | 6（9.2%） | - | - |
| 3-4W | 6（9.2%） | 3（4.6%） | 4（6.2%） | - | - |
| 5-8W | 1 （1.5%） | 2（3.1%） | 1（1.5%） | - | - |
| 9-12W | 0（0%） | 0（0%） | 1（1.5%） | - | - |
| Total | 16.9% | 15.4% | 18.5% | 0.219 | 0.896 |

Data are shown as n (%).

^#^*P* value represents a comparison between three groups at the same point in time.
